# The Burden of Chronic Disease: The Future is Prevention: Introduction to Dr. James Marks' presentation, “The Burden of Chronic Disease and the Future of Public Health”

**Published:** 2004-03-15

**Authors:** George E. Hardy

**Affiliations:** Association of State and Territorial Health Officials

Chronic diseases impose an enormous financial and societal burden on the United States. According to the Centers for Disease Control and Prevention (CDC), chronic diseases today account for 70% of the deaths of all Americans and 75% of this country’s annual health care costs. Unless we take steps now to deal effectively with chronic diseases, our nation is headed for a serious financial and quality-of-life crisis. Among the contributing factors to this crisis are the aging of our population; increases in obesity, particularly among adolescents; and the tragedy of tobacco addiction.

No one speaks with more passion, conviction, and vision about the need to address this pending crisis than Dr. James Marks, director of the CDC’s National Center for Chronic Disease Prevention and Health Promotion. As he demonstrates so clearly in his presentation, "The Burden of Chronic Disease and the Future of Public Health," public health prevention programs can, with real societal and political will, substantially reduce or even prevent the burden of many major chronic disease conditions.

His presentation makes a strong case for moving from a palliative medical model to a prevention-based approach. He argues most persuasively that preventing chronic diseases can provide Americans with a better quality of life, reduce unnecessary medical costs and lost productivity, and strengthen our national economy.

Funding for research and medical advances alone will alleviate neither the cost nor the suffering of individuals faced with a chronic disease. Without concomitant investments in public health prevention and control efforts to implement the prevention research results we already have, rising health care costs and preventable deaths will continue. Americans deserve a national commitment to translating critically important research findings into practical public health solutions.

Despite limited resources, state public health agencies are working to create healthy opportunities for all Americans by partnering with public, private, and voluntary organizations to ensure a strategic application of known prevention successes to chronic disease prevention programming and resources. Public health professionals are also working with Medicaid agencies to develop clinical assistance programs and to explore policy changes that could benefit individuals and communities by more effectively targeting scarce prevention resources. Imagine what could be done if a real national commitment were made to provide the resources necessary to truly address the challenges and opportunities of an aging population and the risk factors attendant to chronic diseases.

Because of the increasing burden of chronic diseases, the United States faces a potential financial and health care crisis of unparalleled proportion. We must not lose this opportunity to do whatever we can to reduce the costly and unnecessary burden of chronic disease that will continue to fuel that crisis. As health professionals, it is incumbent upon us to join Dr. Marks in ensuring that our nation’s political leaders and the citizens they represent better understand the profound burden of chronic disease and the positive efforts that need to be taken now to reduce that burden. Our nation deserves no less.

